# COVID-19-Related Changes in Potential Risk Factors for Elder Mistreatment Reported by Caregivers of Older Adults

**DOI:** 10.1093/geroni/igaa057.3443

**Published:** 2020-12-16

**Authors:** Lena Makaroun, Scott Beach, Tony Rosen, Ann-Marie Rosland

**Affiliations:** 1 University of Pittsburgh School of Medicine, Pittsburgh, Pennsylvania, United States; 2 University of Pittsburgh, Pittsburgh, Pennsylvania, United States; 3 Weill Cornell Medicine / NewYork-Presbyterian Hospital, New York, New York, United States

## Abstract

In previous studies, caregiver (CG) stress, substance use, poor physical health, poor mental health, financial problems, and social isolation have been associated with increased risk of elder mistreatment (EM) for older care recipients (CR). This study aimed to assess how the COVID-19 pandemic has impacted these CG-related risk factors for EM in a community sample of CGs. A non-probability sample of 433 CGs caring for adult CRs age ≥60 years with physical (76%), cognitive (34%) and mental health (14%) conditions completed a survey on COVID-19 impacts in April-May 2020. CGs had mean age 61 (range 21 – 91), were 75% female and 92% non-Hispanic White. Over 40% of CGs reported doing worse financially since COVID-19. Compared to before COVID-19, 15% reported drinking more alcohol and 64% reported somewhat or greatly increased feelings of social isolation and loneliness. CGs reported that COVID-19 had made caregiving more physically (18.7%), emotionally (48.5%) and financially (14.5%) difficult, interfered with their own healthcare (19%), and led to family conflict over caring for CR (13.2%). Younger CGs (age <65) and those with annual income <$50,000 were more likely to report negative COVID-19 impacts. This study suggests CGs of older adults may be experiencing increased stress, alcohol use, social isolation and negative impacts on their own health and financial situation. Healthcare and social service providers should assess for these EM risk-factors in caregivers and connect them and their care recipients with resources and services to address these stressors to reduce risk of EM during the COVID-19 pandemic.

